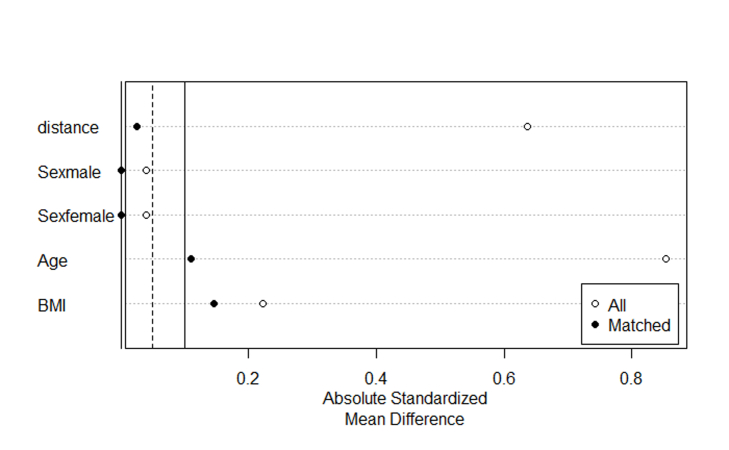# The impact of statins on pulmonary embolism severity—a retrospective data analysis

**DOI:** 10.1016/j.rpth.2025.102982

**Published:** 2025-07-22

**Authors:** Paul Gressenberger, Bettina Wachmann, Andrea Borenich, Gudrun Pregartner, Lisa Moser, Nikolaus Schreiber, Johannes Schmid, Ewald Kolesnik, Günther Silbernagel, Reinhard B. Raggam, Marianne Brodmann, Thomas Gary

**Affiliations:** 1 Department of Dermatology and Venereology, Medical University of Graz, Graz, Austria; 2 Division of Angiology, Department of Internal Medicine, Medical University of Graz, Graz, Austria; 3 Institute for Medical Informatics, Statistics and Documentation, Medical University of Graz, Graz, Austria; 4 Department of Internal Medicine, Medical University of Graz, Graz, Austria; 5 Division of Anaesthesiology and Intensive Care Medicine, Medical University of Graz, Graz, Austria; 6 Division of General Radiology, Department of Radiology, Medical University of Graz, Graz, Austria; 7 Division of Cardiology, Department of Internal Medicine, Medical University of Graz, Graz, Austria

**Keywords:** pulmonary embolism, pulmonary embolism severity, risk assessment, retrospective studies, statins

## Abstract

**Background:**

Recent studies have demonstrated decreased rates of venous thrombotic events, including pulmonary embolism (PE), in patients taking statins. It, however, remains elusive whether statins could also impact PE severity.

**Objectives:**

To investigate a potential association between statin use and the severity of PE in a retrospective cohort.

**Methods:**

We performed a retrospective data analysis of patients with PE confirmed by computed tomography pulmonary angiography between January 1, 2010, and December 31, 2019, at the University Hospital Graz, Austria. PE severity was assessed based on the 2019 European Society of Cardiology guidelines.

**Results:**

Of 1590 patients analyzed, 235 (14.7%) were statin users. Statin users were significantly older than nonusers (median, 74 years [IQR, 66-80] vs 67 years [IQR, 52-78]; *P* < .001) and had a higher body mass index (BMI kg/m^2^; median, 27.4 [IQR, 24.7-30.7] vs 26.2 [IQR, 23.6-29.7]; *P* = .001). Statin users had a significantly higher prevalence of comorbidities, including kidney insufficiency, arterial hypertension, diabetes, hyperlipidemia, atherosclerotic cardiovascular disease (all *P* < .001), and heart failure (*P* = .006), while the nonstatin group had a higher prevalence of cancer (29.6% vs 14.0%; *P* = .04). Our study revealed a significantly smaller proportion of low-risk PE in statin users compared with nonstatin users (12.3% vs 19.9%; *P* = .006). After matching the groups based on sex (male and female), age, and BMI (kg/m^2^), no significant differences in PE severity were found.

**Conclusion:**

Statin use was not associated with PE severity. The smaller proportion of low-risk PE in statin users is likely attributable to their older age, higher BMI, and comorbidities.

## Essentials

• Statins may influence the severity of PE.
• We analyzed 1590 PE patients at a single academic center.
• Statin users had more comorbidities but no evidence of milder PE.
• Statin use did not impact PE severity or survival outcomes.

## 1 Introduction

Elevated cholesterol levels are strongly associated with the development of atherosclerotic cardiovascular diseases (ASCVDs) such as stroke, coronary heart disease, or peripheral artery disease [1]. Therefore, the European Society of Cardiology (ESC) recommends measuring cholesterol levels as part of assessing cardiovascular risk (CVR). Management strategies to lower CVR include lifestyle changes like healthy diet and exercise, or, if necessary, for patients at increased risk of ASCVD, statin therapy is recommended [2]. Statins are primarily known for their cholesterol–lowering effects and have provided outstanding contributions in the prevention of ASCVD [3]. Besides, statins have antiinflammatory properties, as well as potential effects on blood clotting and endothelial function improvement [4]. Recent studies have demonstrated decreased rates of venous thrombotic events (VTE), such as pulmonary embolism (PE), in patients taking statins, which may be linked to these mechanisms [5,6]. It, however, remains elusive whether these protective effects could also impact PE severity. The severity of PE can vary from mild cases that might not cause significant symptoms to life-threatening conditions, especially when massive PE is present, causing significant morbidity and mortality [7,8]. The current ESC guidelines for the management of acute PE provide a structured approach to assess PE-related severity based on different risk categories [9,10]. Using this classification, we aimed to investigate a potential association between statin use and PE severity in a single-center cohort through retrospective data analysis.

## 2 Methods

We performed a retrospective analysis of patients diagnosed with PE at the University Hospital Graz, Austria, between January 1, 2010, and December 31, 2019. Cases were initially identified via diagnostic codes from the hospital’s electronic medical records system. To ensure diagnostic accuracy, all corresponding computed tomography pulmonary angiography (CTPA) reports were manually reviewed to confirm the presence of acute PE and to exclude chronic PE. Only inpatient cases were included in the study. While most patients were diagnosed in the emergency department, a detailed breakdown by location (eg, emergency department, inpatient ward, or outpatient clinic) was not systematically recorded. We then identified those who were on concomitant statin therapy at the time of PE diagnosis. Statin use was determined based on active medication lists and discharge summaries at the time of hospital admission and was confirmed through manual chart review. Patients in whom statin therapy was newly initiated during hospitalization were not included in the statin group. Following the 2019 ESC guidelines for the diagnosis and management of acute PE [9], we conducted a severity assessment and classified patients into 4 categories: low risk (LR), intermediate LR (IML), intermediate high risk (IMH), and high risk. According to the ESC criteria, patients with hemodynamic instability were considered high risk, which was defined by one of the following clinical presentations: cardiac arrest, obstructive shock (systolic blood pressure [BP] < 90 mm Hg or the need for vasopressors to maintain a BP ≥ 90 mm Hg), or persistent hypotension (systolic BP < 90 mm Hg or a drop of ≥ 40 mm Hg for >15 minutes not due to new-onset arrhythmia, hypovolemia, or sepsis).

The simplified PE Severity Index score, elevated cardiac biomarkers (troponin and/or N-terminal pro B-type natriuretic peptide), and the presence of right ventricular dysfunction (RVD) on CTPA or transthoracic echocardiography (TTE) were used to provide prognostic information for categorizing patients as LR, intermediate risk, or high risk. In our study, the simplified PE Severity Index score was calculated based on age, history of cancer, chronic cardiopulmonary disease (CCPD), oxygen saturation, systolic BP, and heart rate using the initial vital signs recorded in the emergency department report at the time of PE diagnosis. RVD was assessed based on imaging findings from either CTPA or TTE, as available. While TTE was performed in a subset of patients, the exact number of TTEs was not systematically recorded. For the purposes of ESC risk classification, the presence or absence of RVD was documented as a binary variable (yes/no), based on the available imaging data. At our hospital, CTPA and RVD assessment by specialized radiologists is part of standard care. In select cases, point-of-care echocardiography was performed to assess RVD. Computed tomography criteria for RVD included a right-to-left ventricular diameter ratio of >1, bulging of the interventricular septum, and reflux of contrast media into the inferior vena cava and hepatic veins. Echocardiographic RVD assessments were performed on a case-by-case basis by treating physicians in our acute care facilities. RVD was considered present if it was documented on either CTPA or TTE. In cases where both modalities were available and results differed, the presence of RVD on either modality led to classification as “RVD yes,” to ensure consistent risk stratification across the cohort.

Laboratory data were collected within 1 day prior to or after PE diagnosis. Comorbidities were identified using International Classification of Diseases, 10th Revision codes (Supplementary Table 1) and included if documented within a 7-day window before or after PE diagnosis. The selected comorbidities were chosen based on their clinical relevance to PE and statin-related patient profiles. Cancer was also identified using International Classification of Diseases, 10th Revision codes (Supplementary Table 2). Only patients with a documented history of cancer, defined as a diagnosis made within 2 years prior to the PE event, were included to ensure temporal consistency. All major cancer types were considered.

To capture both short-term and long-term outcomes, all-cause mortality was evaluated at 2 predefined time points: 30 days and 2 years after the diagnosis of PE. Vital status at each time point was determined using hospital records and national registry data from Statistics Austria. The study protocol was approved by the Ethics Committee of the Medical University of Graz (EK 34-239 ex 21/22).

### 2.1 Statistical analysis

Patient characteristics were summarized using the median and IQR for continuous data and absolute and relative frequencies for categorical data. Group comparisons were conducted with the Mann–Whitney *U*-test, Pearson’s chi-squared test, or Fisher’s exact test, as appropriate. Logistic regression analyses were applied to assess predictors for LR PE (vs intermediate- and high-risk pooled), while Cox regression analyses were used to evaluate 30-day and 2-year survival rates. Results are presented as odds ratios (ORs) or hazard ratios (HR) with the corresponding 95% CIs. Both univariable analyses and those adjusted for sex, age, and BMI (kg/m^2^) were conducted. Additionally, as a sensitivity analysis, statin and nonstatin users were propensity score-matched by sex, age, and BMI in a 1:2 ratio using a nearest neighbor algorithm, followed by univariable regression analyses in the matched dataset (Supplementary Figure). A *P* value of ≤ .05 was considered statistically significant. All statistical analyses were conducted using R version 4.4.1 (R Foundation for Statistical Computing).

## 3 Results

### 3.1 Patient characteristics

Of 1778 patients screened, 1590 were included in the final analysis after risk stratification. A total of 188 patients were excluded from the final analysis due to missing key clinical data required for ESC risk stratification, such as oxygen saturation, BP, or troponin values, which made a reliable classification of PE severity impossible. Statin users (*n* = 235, 14.8%) were significantly older (median, 74.0 [IQR, 66.0-80.0] vs 67.0 [IQR, 52.0-78.0] years; *P* < .001) and had a higher BMI (median, 27.4 [IQR, 24.7-30.7] vs 26.2 [IQR, 23.6-29.7]; *P* = .001) compared with nonusers. They also had a higher prevalence of comorbidities, including kidney insufficiency, hypertension, diabetes, hyperlipidemia, and ASCVD (all *P* < .001), as well as heart failure (*P* = .006), while cancer was more common in nonstatin users (19.6% vs 14.0%; *P* = .04). Laboratory parameters such as cholesterol, high-density lipoprotein, low-density lipoprotein, and triglycerides did not differ significantly between groups; however, creatinine (median, 1.03 [IQR, 0.85-1.27] vs 0.97 [IQR, 0.82-1.16] mg/dL; *P* = .003), troponin high-sensitive (median, 17.0 [IQR, 8.0-40.0] vs 24.3 [IQR, 12.0-59.0] ng/L; *P* < .001), and HbA1c levels (median, 42.6% [IQR, 38.0%-47.8%] vs 37.7% [IQR, 35.3%-43.0%]; *P* = .007) were higher in statin users, although the low number of available measurements in parts of the cohort limits the interpretability of these findings. The most commonly prescribed statin was simvastatin (61.7%), followed by atorvastatin (26.0%), rosuvastatin (6.8%), pravastatin (3.8%), and fluvastatin (1.7%; Table 1).

**Table 1:** Baseline characteristics.

Characteristic	Overall *N* = 1590	Nonstatin users *n* = 1355	Statin users *n* = 235	*P* value^a^
Sex				.61
Male	835.0 (52.5)	708.0 (52.3)	127.0 (54.0)	
Female	755.0 (47.5)	647.0 (47.7)	108.0 (46.0)	
Age at PE diagnosis	68.0 (54.0, 78.0)	67.0 (51.0, 78.0)	74.0 (66.0, 80.0)	< .001
BMI (kg/m^2^)	26.5 (23.7, 29.8)	26.2 (23.6, 29.7)	27.4 (24.7, 30.7)	.001
*Missing*	159	140	19	
Cholesterol, mg/dL	175.5 (140.0, 203.0)	179.0 (145.0, 206.0)	168.0 (137.0, 192.0)	.09
*Missing*	1380	1178	202	
HDL, mg/dL	45.0 (37.0, 58.0)	45.0 (37.0, 58.0)	45.5 (36.0, 53.5)	.61
*Missing*	1451	1240	211	
LDL, mg/dL	100.5 (80.0, 126.0)	102.0 (82.0, 130.0)	94.0 (76.0, 112.0)	.09
*Missing*	1462	1248	214	
Triglycerides, mg/dL	115.0 (84.0, 168.0)	113.0 (83.0, 157.0)	134.0 (86.0, 192.0)	.26
*Missing*	1381	1179	202	
D-dimer, μg/mL	4.3 (2.1, 9.9)	4.3 (2.1, 9.8)	4.7 (2.4, 10.3)	.52
*Missing*	429	362	67	
Creatinine, mg/dL	0.98 (0.82, 1.17)	0.97 (0.82, 1.16)	1.03 (0.85, 1.27)	.003
*Missing*	42	35	7	
Hs troponin T, ng/L	19.0 (8.0, 41.0)	17.0 (8.0, 40.0)	24.3 (12.0, 59.0)	< .001
*Missing*	*411*	*374*	*37*	
NT-proBNP, pg/mL	627.0 (164.0, 3026.0)	609 (156.0, 3489.0)	715 (245.5, 2224.0)	.36
*Missing*	*976*	*854*	*122*	
HBA1C, mmol/mol	38.8 (36.0, 45.0)	37.7 (35.3, 43.0)	42.6 (38.0, 47.8)	.007
*Missing*	*1487*	*1276*	*211*	
Nicotine abuse	151.0 (9.5)	128.0 (9.4)	23.0 (9.8)	.896
Kidney insufficiency	249.0 (15.6)	191.0 (14.1)	58.0 (24.7)	< .001
Arterial hypertension	621.0 (39.1)	466.0 (34.4)	155 (66.0)	< .001
Diabetes	117.0 (7.4)	75.0 (5.5)	42.0 (17.9)	< .001
Hyperlipidemia	130.0 (8.2)	61.0 (4.5)	69.0 (29.4)	< .001
Heart failure	104.0 (6.5)	79.0 (5.8)	25.0 (10.6)	.006
Cancer	299.0 (18.8)	266.0 (19.6)	33.0 (14.0)	.04
ASCVD	312.0 (19.6)	208.0 (15.4)	104.0 (44.3)	< .001
CPD	214.0 (13.5)	175.0 (12.9)	39.0 (16.6)	.127
**Statin_type**				> .999
Atorvastatin	61.0 (26.0)	-	61.0 (26.0)	
Fluvastatin	4.0 (1.7)	-	4.0 (1.7)	
Pravastatin	9.0 (3.8)	-	9.0 (3.8)	
Rosuvastatin	16.0 (6.8)	-	16.0 (6.8)	
Simvastatin	145.0 (61.7)	-	145.0 (61.7)	

Data are presented as *n* (%) or median (Q1, Q3).

ASCVD, atherosclerotic cardiovascular disease; BMI, body mass index; CPD, chronic pulmonary disease; HDL, high-density lipoprotein; Hs, high-sensitive; HBA1C, hemoglobin A1c; LDL, low-density lipoprotein; NT-proBNP, N-terminal prohormone of brain natriuretic peptide; PE, pulmonary embolism.

a Pearson’s chi-squared test; Wilcoxon rank-sum test; Fisher’s exact test.

To provide an overview of the key prognostic factors relevant to PE risk stratification, we summarized selected clinical variables in Table 2. Some of these variables, such as cancer and CCPD, are also listed in Table 1, as they are clinically relevant for both descriptive and risk classification purposes. CCPD was defined as the presence of either heart failure or chronic pulmonary disease, based on the corresponding parameters reported in Table 1. Statin users had a higher prevalence of CCPD (25.1% vs 17.6%; *P* = .007), while heart rate ≥ 110 bpm (14.1% vs 21.6%; *P* = .01), hemodynamic instability (1.7% vs 5.2%; *P* = .037), and a history of cancer (14.0% vs 19.6%; *P* = .04) were significantly less frequent in this group. Other variables, including age ≥80 years, systolic BP < 100 mm Hg, oxygen saturation <90%, and RVD, showed no statistically significant differences between groups.

**Table 2:** Indicators of risk.

Characteristic	Overall (*N* = 1590) *n* (%)	No statin use (*n* = 1355) *n* (%)	Statin use (*n* = 235) *n* (%)	*P* value
Age ≥80 y	299 (18.8)	249 (18.4)	50 (21.3)	.29
History of cancer	299 (18.8)	266 (19.6)	33 (14.0)	.04
CCPD	298 (18.7)	239 (17.6)	59 (25.1)	.007
Heart rate ≥ 110 bpm	296 (20.5)	266 (21.6)	30 (14.1)	.01
*Missing*	*145*	*123*	*22*	
Systolic BP < 100 mm Hg	78 (5.62)	64 (5.4)	14 (6.6)	.478
*Missing*	*202*	*178*	*24*	
O_2_ saturation <90%	166 (14.8)	139 (14.5)	27 (16.5)	.515
*Missing*	*468*	*397*	*71*	
RVD	493 (31.3)	420 (31.3)	73 (31.5)	.959
*Missing*	*16*	*13*	*3*	
Hemodynamic instability	55 (4.69)	52 (5.24)	3 (1.67)	.037
*Missing*	*418*	*363*	*55*	

Cardiac biomarkers (high-sensitive troponin T and N-terminal prohormone of brain natriuretic peptide), also relevant for risk stratification, are presented in Table 1.

BP, blood pressure; CCPD, chronic cardiopulmonary disease, including heart failure and chronic pulmonary disease, from Table 1; RVD, right ventricular dysfunction.

Among the patients with a history of cancer (*n* = 299, 18.8%), the most frequently documented tumor types were hepato-pancreato–biliary cancer (23.4%) and genitourinary cancer (20.4%), followed by metastases, which were present in 47.2% of all cancer cases. Hematologic malignancies (15.4%), breast cancer (9.7%), brain tumors (9.4%), esophageal cancer (6.0%), and colorectal cancer (5.7%) were also commonly observed. Hepatocellular carcinoma (1.3%) and melanoma (1.67%) were rare overall. Lung cancer was documented in only 2 patients, both of whom were nonstatin users (Table 3).

**Table 3:** Distribution of cancer types among patients with a history of cancer.

Cancer type	Overall (*N* = 299) *n* (%)	No statin use (*n* = 266) *n* (%)	Statin use (*n* = 33) *n* (%)	*P* value^a^
Brain tumor	28 (9.4)	23 (8.6)	5 (15.2)	.21
Breast cancer	29 (9.7)	25 (9.4)	4 (12.1)	.54
Colorectal cancer	17 (5.7)	16 (6.0)	1 (3.0)	.70
Esophageal cancer	18 (6.0)	15 (5.6)	3 (9.1)	.43
Genitourinary cancer	61 (20.4)	55 (20.7)	6 (18.2)	.737
Gynecological cancer	9 (3.0)	8 (3.0)	1 (3.0)	> .999
Hematologic	46 (15.4)	42 (15.8)	4 (12.1)	.58
Hepatocellular carcinoma	4 (1.3)	3 (1.1)	1 (3.0)	.375
Hepato-pancreato-biliary cancer	70 (23.4)	63 (23.7)	7 (21.2)	.75
Lung cancer	2 (0.7)	2 (0.8)	0 (0)	> .999
Melanoma	5 (1.67)	5 (1.88)	0	> .999
Metastases	141 (47.2)	129 (48.5)	12 (36.4)	.188

a Pearson’s chi-squared test; Fisher’s exact test.

### 3.2 Outcomes and mortality

**Figure 1:**
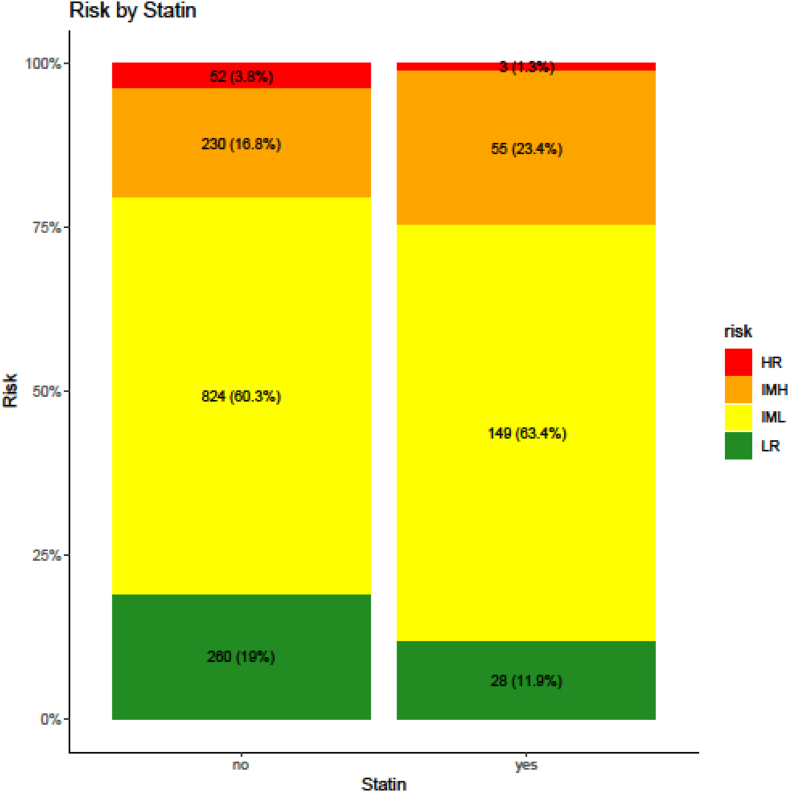
Unadjusted distribution of pulmonary embolism severity categories: low risk (LR), intermediate low risk (IML), intermediate high risk (IMH), and high risk (HR) among statin users and nonusers.

Statin users had a significantly lower proportion of LR PE (12.39% vs 19.9%; *P* = .006), with a higher proportion of both IML and IMH cases (63.0% vs 59.3% for IML and 23.4% vs 17.0% for IMH). Finally, the high-risk category was lower in statin users (1.3% vs 3.8%; Figure 1 and Table 4). Overall mortality was comparable between statin users and nonusers (47.2% vs 41.2%; *P* = .08). Similarly, no significant differences were observed for 30-day mortality (4.3% vs 5.2%; *P* = .526) or 2-year mortality (22.1% vs 24.0%; *P* = .537; Table 4).

**Table 4:** Risk distribution and mortality outcomes.

Characteristic	Overall *N* = 1590 *n* (%)	Nonstatin users *n* = 1355 *n* (%)	Statin users *n* = 235 *n* (%)	*P* value
**Risk**				.02^a^
LR	298 (18.7)	269 (19.9)	29 (12.3)	
IML	952 (59.9)	804 (59.3)	148 (63.0)	
IMH	285 (17.9)	230 (17.0)	55 (23.4)	
HR	55 (3.5)	52 (3.8)	3 (1.3)	
**Low risk**				.006^b^
LR	298 (18.7)	269 (19.9)	29 (12.3)	
No LR	1292 (81.3)	1086 (80.1)	206 (87.7)	
Overall mortality	669 (42.1)	558 (41.2)	111 (47.2)	.08^b^
30-d	81 (5.09)	71 (5.24)	10 (4.26)	.526^b^
2-y	377 (23.7)	325 (24.0)	52 (22.1)	.537^b^

HR, high risk; IMH, intermediate high risk; IML, intermediate low risk; LR, low risk.

a Wilcoxon rank-sum test.

b Pearson’s chi-squared test.

### 3.3 Regression analyses

Logistic regression analysis showed that statin use was not independently associated with an LR PE classification after adjustment for age, sex, and BMI (kg/m^2^) (OR, 0.92; 95% CI, 0.58-1.46; *P* = .729). Age, however, was a significant predictor, with older patients less likely to be LR (OR, 0.96 per year; 95% CI, 0.95-0.97; *P* < .001).

**Figure 2:**
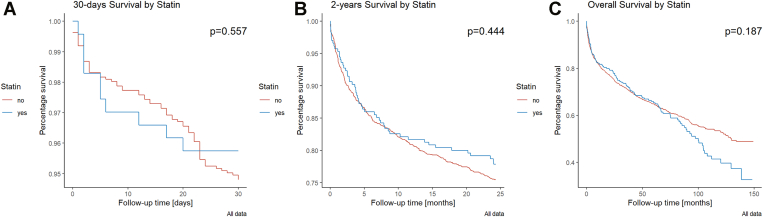
Survival analyses using Kaplan–Meier estimates.

Cox regression analysis revealed no significant effect of statins on 30-day (HR, 0.85; 95% CI, 0.42-1.72; *P* = .647) or 2-year survival (HR, 0.86; 95% CI, 0.63-1.17; *P* = .346; Figure 2). Older age increased mortality risk (HR, 1.03 per year; 95% CI, 1.02-1.04; *P* < .001), while higher BMI (kg/m^2^) reduced it (HR, 0.94; 95% CI, 0.92-0.97; *P* < .001; numbers are for 2-year mortality).

### 3.4 Matched analysis

A matched analysis was performed with a 1:2 ratio for statin vs nonstatin users (*n* = 216 vs *n* = 432), based on sex, age, and BMI (kg/m^2^), to account for these important confounders. After matching, the statin group had a higher prevalence of kidney insufficiency (*P* = .025), hypertension, diabetes, ASCVD, and hyperlipidemia (all *P* < .001), but a lower prevalence of cancer (14.8% vs 21.3%; *P* = .048).

### 3.5 Matched outcomes and mortality

In the matched cohort (*n* = 648), no significant differences were found in overall risk stratification (LR vs IML vs IMH vs high risk; *P* = .50) or the proportion of LR patients (13.0% for statin vs 13.2% for nonstatin users; *P* = .93). Mortality rates were similar (47.2% in statin vs 48.6% in nonstatin users; *P* = .739), with a median time to death of 806 (IQR, 121-2001) days in the statin group vs 553 (IQR, 102-1507) days in the nonstatin group.

### 3.6 Regression analyses

Logistic regression showed no significant association between statin use and LR PE classification (OR, 0.98; 95% CI, 0.60-1.58; *P* = .93) after matching. Cox regression revealed no significant impact of statin use on 30-day (HR, 0.86; 95% CI, 0.39-1.88; *P* = .706) or 2-year survival (HR, 0.82; 95% CI, 0.59-1.15; *P* = .26).

## 4 Discussion

Statins have sparked interest due to their potential impact on VTE, including PE [11,12]. While statins are primarily recognized for their role in protecting patients from ASCVD and reducing CVR, emerging research has suggested additional mechanisms that might influence the risk of VTE [13,14]. It, however, remains elusive whether these effects could also impact the severity of PE. In this context, we aimed to investigate the association between statin use and PE severity.

Our results indicate that statin use was not significantly associated with the severity of PE or survival outcomes. Although statin users initially had a significantly lower proportion of LR PE, this difference disappeared after adjusting for confounders, including sex, age, and BMI. The higher prevalence of comorbidities, including kidney insufficiency, arterial hypertension, diabetes, hyperlipidemia, heart failure, and ASCVD in statin users, coupled with their older age and higher BMI, likely contributed to their higher risk categorization [[15], [16], [17], [18]]. In contrast, the nonstatin group had a higher prevalence of cancer, a known contributor to increased PE severity [19,20]. Although some studies have reported potential anticancer effects of statins [21], our study was not designed to explore this question. The lower prevalence of cancer in the statin group should, therefore, be interpreted with caution. This difference may be related to confounding, selection bias, or other differences in patient characteristics, rather than a direct effect of statin use. While VTE and ASCVD are distinct conditions with different pathophysiology, shared mechanisms such as endothelial dysfunction and inflammation may provide a basis for statins’ potential role in VTE prevention or mitigation [16,17,22]. Some studies suggest a dose-dependent protective effect of statins on VTE, which we could not evaluate in this study [23,24]. Additionally, while earlier research reported reduced 30-day mortality in statin users with PE [25], our study found no significant differences in mortality at 30 days or 2 years. Our retrospective design introduces limitations, including potential selection bias and unmeasured confounders. Despite adjusting for key factors such as age, sex, and BMI, we could not assess all VTE-related risk factors, idiopathic PE cases, or the impact of concomitant medications and the duration of statin use before diagnosis. Additionally, the severity assessment may have limitations, such as automatically classifying patients with comorbidities like cancer into intermediate-risk categories, even with minimal PE involvement. Despite a meaningful sample size, the study may be underpowered to detect subtle associations between statin use and PE severity, especially given the heterogeneity of patient characteristics and the missing data for several key variables, such as cholesterol levels and cardiac biomarkers.

In conclusion, statin use was not associated with PE severity or mortality in our cohort. Nevertheless, the observed trends and shared mechanisms between VTE and ASCVD highlight the need for further research to address potential antithrombotic properties of statins.

## Figures and Tables

**Supplemental Figure:**